# Artificial Intelligence for Skin Cancer Detection: Scoping Review

**DOI:** 10.2196/22934

**Published:** 2021-11-24

**Authors:** Abdulrahman Takiddin, Jens Schneider, Yin Yang, Alaa Abd-Alrazaq, Mowafa Househ

**Affiliations:** 1 Department of Electrical and Computer Engineering Texas A&M University College Station, TX United States; 2 College of Science and Engineering Hamad Bin Khalifa University Doha Qatar

**Keywords:** artificial intelligence, skin cancer, skin lesion, machine learning, deep neural networks

## Abstract

**Background:**

Skin cancer is the most common cancer type affecting humans. Traditional skin cancer diagnosis methods are costly, require a professional physician, and take time. Hence, to aid in diagnosing skin cancer, artificial intelligence (AI) tools are being used, including shallow and deep machine learning–based methodologies that are trained to detect and classify skin cancer using computer algorithms and deep neural networks.

**Objective:**

The aim of this study was to identify and group the different types of AI-based technologies used to detect and classify skin cancer. The study also examined the reliability of the selected papers by studying the correlation between the data set size and the number of diagnostic classes with the performance metrics used to evaluate the models.

**Methods:**

We conducted a systematic search for papers using Institute of Electrical and Electronics Engineers (IEEE) Xplore, Association for Computing Machinery Digital Library (ACM DL), and Ovid MEDLINE databases following the Preferred Reporting Items for Systematic Reviews and Meta-Analyses Extension for Scoping Reviews (PRISMA-ScR) guidelines. The studies included in this scoping review had to fulfill several selection criteria: being specifically about skin cancer, detecting or classifying skin cancer, and using AI technologies. Study selection and data extraction were independently conducted by two reviewers. Extracted data were narratively synthesized, where studies were grouped based on the diagnostic AI techniques and their evaluation metrics.

**Results:**

We retrieved 906 papers from the 3 databases, of which 53 were eligible for this review. Shallow AI-based techniques were used in 14 studies, and deep AI-based techniques were used in 39 studies. The studies used up to 11 evaluation metrics to assess the proposed models, where 39 studies used accuracy as the primary evaluation metric. Overall, studies that used smaller data sets reported higher accuracy.

**Conclusions:**

This paper examined multiple AI-based skin cancer detection models. However, a direct comparison between methods was hindered by the varied use of different evaluation metrics and image types. Performance scores were affected by factors such as data set size, number of diagnostic classes, and techniques. Hence, the reliability of shallow and deep models with higher accuracy scores was questionable since they were trained and tested on relatively small data sets of a few diagnostic classes.

## Introduction

### Background

Skin cancer is the most common cancer type that affects humans [1]. Melanoma and nonmelanoma are the two main types of skin cancer [2]. Nonmelanoma is of lesser concern since it usually can be cured by surgery and is nonlethal. Melanoma, however, is the most dangerous skin cancer type, with a high mortality rate, although it represents less than 5% of all skin cancer cases [1]. The World Health Organization (WHO) estimated 132,000 yearly melanoma cases globally. In 2015, 60,000 cases caused death [2].

Traditional methods of early detection of skin cancer include skin self-examination and skin clinical examination (screening) [3]. However, skin self-examination, where the patient or a family member notices a lesion, is a random method as people might overreact or underact. In addition, clinical examination using expensive, specialized medical tools, such as a dermoscope, microspectroscopy, and laser-based tools, requires training, effort to operate, time, and regular follow-ups [4]. Thus, patients have started using mobile technologies, such as smartphones, to share images with their doctors to get faster diagnoses. However, sharing images over the internet may compromise privacy. Worse yet, the image quality may not be sufficient, which may lead to inaccurate diagnoses. With evolvement, artificial intelligence (AI), which is the human-like intelligence exhibited by trained machines [5], has become so pervasive that most humans interact with AI-based tools daily, which assists physicians in decision making and decreases the decision variations among physicians. It is worth mentioning that even with the presence of such AI technologies, the role of an expert dermatologist is vital for diagnosis and treatment.

The focus of this review is on the use of AI as a tool that helps in the process of skin cancer diagnostics. Herein, AI-based skin cancer diagnostic tools use either shallow or deep AI methodologies. Both involve customizing computer algorithms through a process called training to learn from data formed by predefined features. The difference is that shallow methods tend to not use multilayer neural networks at all or use such networks limited to a minimum of layers [6]. In contrast, deep methodologies involve training large, deep multilayer neural networks with many hidden layers, typically ranging from dozens to hundreds [7].

### Research Problem

**Figure 1:**
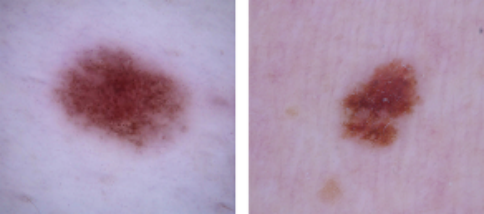
Similarity of normal lesion (left) and melanoma (right).

Detecting skin cancer can be challenging, time consuming, and relatively expensive [4]. For example, Figure 1 shows two lesions that superficially seem identical [8]. However, the left image is of a normal benign lesion, whereas the right image shows a melanoma lesion. As AI technologies are becoming smarter and faster [5], it is hardly surprising that they are being used to assist in diagnosing skin cancer and suggesting courses of action. This is due to the fact that AI-based methods are considered to be relatively cheap, easy to use, and accessible [5]. Thus, they offer the potential to overcome the issues inherent in the aforementioned existing skin cancer detection methods. However, as the literature on the medical use of AI quickly grows and continues to report findings using incompatible performance metrics, direct comparison between prior work becomes more challenging and threatens to hamper future research. This study seeks to address this issue by performing a rigorous and transparent review of the existing literature. We aim to answer the research question, *What are the existing AI-based tools that are used to detect and classify skin cancer?*

## Methods

This scoping review analyzes papers from different online databases. We defined strict inclusion and exclusion criteria to decide which papers to include. We then grouped the papers by the methodology used and analyzed the ground covered in the papers. Finally, we identified gaps in the literature and discussed how these gaps can be filled by future work. We developed a protocol before commencing the review. To ensure that this scoping review is transparent and replicable, we followed the Preferred Reporting Items for Systematic Reviews and Meta-Analyses Extension for Scoping Reviews (PRISMA-ScR) instructions and guidelines [9].

### Search Strategy

We conducted a systematic search on July 15, 2020. We identified articles from Institute of Electrical and Electronics Engineers (IEEE) Xplore, Association for Computing Machinery Digital Library (ACM DL), and Ovid MEDLINE databases. The terms used for searching the bibliographic databases were identified based on the target population (eg, “skin neoplasms,” “skin cancer,” “skin lesion”), intervention (eg, “artificial intelligence,” “machine learning,” “deep learning”), and outcome (“diagnosis,” “screening,” “detection,” “classification”). We derived the search terms from previous literature studies and reviews. For practical reasons, we did not conduct backward or forward reference list checking, and we also did not contact experts. Multimedia Appendix 1 shows the search strategy used for searching Ovid MEDLINE, where “skin neoplasms,” “artificial intelligence,” “machine learning,” and “deep learning” were used as MESH terms. Multimedia Appendix 1 also shows the search query for IEEE Xplore and ACM DL.

### Study Eligibility Criteria

We included studies fulfilling the following criteria:

- Studies published between January 1, 2009, and July 15, 2020.
- Studies written in English.
- Population: studies discussing only skin cancer. Studies discussing other diseases or forms of cancer were excluded.
- Intervention: studies discussing only AI-based applications. Studies that discussed skin cancer–related applications or systems, including theoretical, statistical, or mathematical approaches, were excluded.
- Studies discussing the specific use of AI for detecting, classifying, or diagnosing skin cancer. Studies discussing only the general use of AI in a clinical setting were excluded.
- Studies proposing a new AI-based method. Case studies, surveys, review or response papers, or papers that reviewed, assessed, analyzed, evaluated, or compared existing methods were excluded.

No restrictions on the country of publication, study design, comparator, or outcomes were enforced.

### Study Selection

Authors Abdulrahman Takiddin (AT) and Alaa Abd-Alrazaq (AA) independently screened the titles and abstracts of all retrieved studies. Following the written protocol, they independently read the full texts of the papers included in this study after reading their titles and abstracts. Any disagreements between both reviewers were resolved by discussion. We assessed the intercoder agreement by calculating the Cohen kappa (κ), which was 0.86 and 0.93 for screening titles and abstracts and for reading full texts, respectively, indicating good agreement.

### Data Extraction

For reliable and accurate data extraction from the included studies, a data extraction form was developed and piloted using eight included studies (Multimedia Appendix 2). The data extraction process was independently conducted by AT and AA. Any disagreements were resolved by discussion with good intercoder agreement (Cohen κ=0.88) between the reviewers.

### Data Synthesis

A narrative approach was used to synthesize the extracted data. Specifically, we first grouped the included studies by diagnostic techniques based on complexity. Then, we discussed the evaluation metrics used in each study. Next, we grouped the studies based on the used evaluation metrics. In addition, we took into consideration the used data set in terms of the number of images, types of images, and number of diseases (diagnostic classes) that the data set contained. We assessed the correlation between the accuracy score and the number of images and diagnostic classes of the data set.

## Results

### Search Results

**Figure 2:**
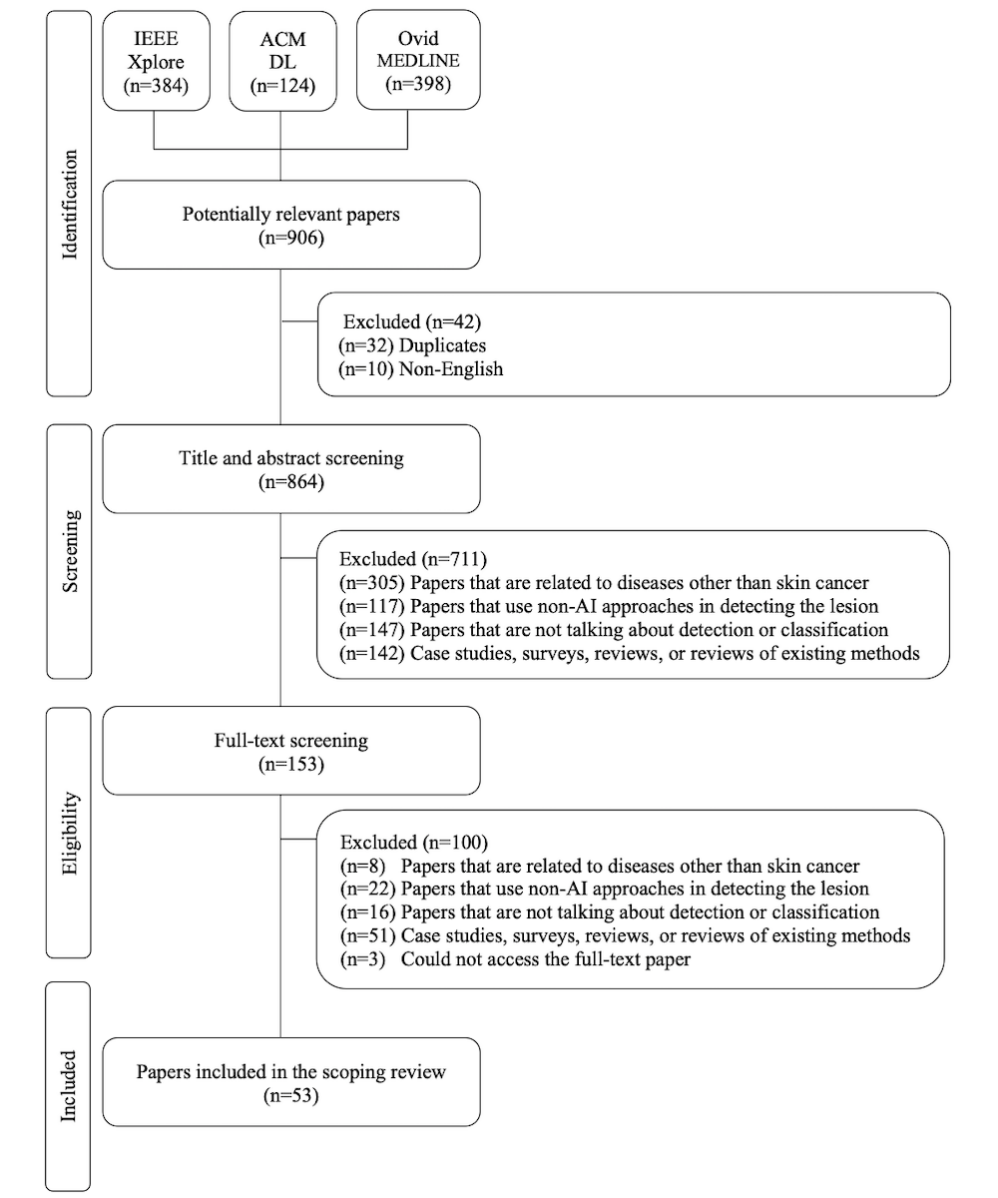
PRISMA approach. ACM DL: Association for Computing Machinery Digital Library; AI: artificial intelligence; IEEE: Institute of Electrical and Electronics Engineers; PRISMA: Preferred Reporting Items for Systematic Reviews and Meta-Analyses.

After searching the 3 online databases, we retrieved a total of 906 studies. We then started excluding papers in three phases. As shown in Figure 2, in the first phase, “identification,” we excluded 42 papers. In the second phase, “screening,” we excluded 711 papers. In the last phase, “eligibility,” we included 153 papers for a full-text review. After reviewing the full text of the papers, we excluded 100 papers. The specific reasons behind excluding the papers in each phase are mentioned in Figure 2. Hence, the total number of included papers in this scoping review was 53.

### Study Characteristics

**Figure 3:**
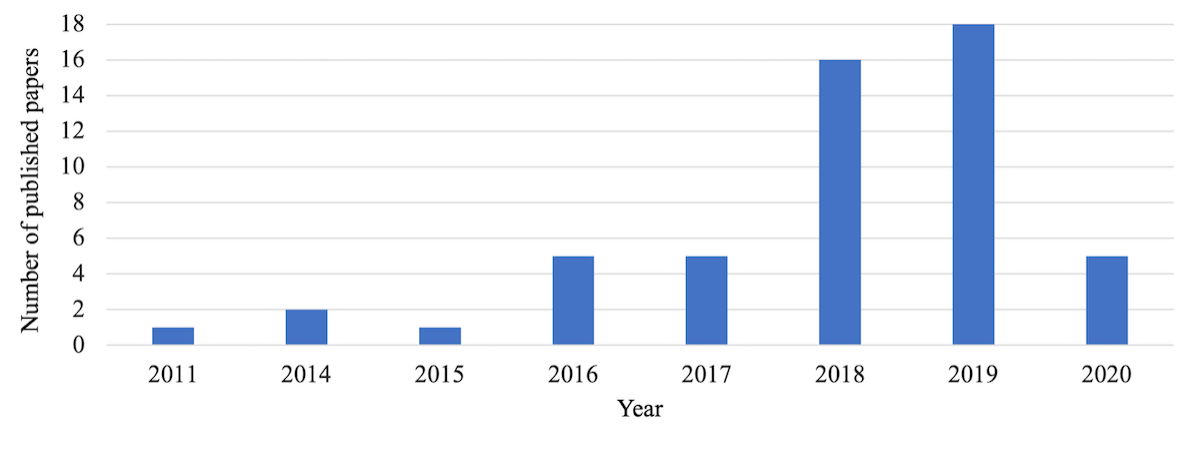
Number of published papers by year.

Table 1 summarizes the characteristics of the selected studies. Figure 3 shows the number of papers published per year: 4 of 53 studies (7.6%) were published before 2016 [10-13], 26 studies (49.1%) were published in 2016, 2017, and 2018 [14-39], and 23 studies (43.4%) were published in 2019 and 2020 [40-62]. Although our selection criteria included papers published between 2009 and July 2020, the oldest published paper included after the full-text review was published in 2011. We observed that the number of papers sharply increased in 2018 and 2019.

**Table 1:** Study characteristics (N=53).

Characteristics		n (%)
**Publication year**		
	Before 2016	4 (7.5)
	2016-2018	26 (49.1)
	2019-2020	23 (43.4)
**Country of publication**		
	The United States	9 (16.9)
	China	6 (11.3)
	India	5 (9.4)
	Poland	3 (5.7)
	New Zealand	2 (3.8)
	Austria	2 (3.8)
	Germany	2 (3.8)
	Bangladesh	2 (3.8)
	Indonesia	2 (3.8)
	Pakistan	2 (3.8)
	Turkey	2 (3.8)
	France	1 (1.9)
	Russia	1 (1.9)
	The United Kingdom	1 (1.9)
	Hong Kong	1 (1.9)
	Iran	1 (1.9)
	Korea	1 (1.9)
	Philippines	1 (1.9)
	Lebanon	1 (1.9)
	Saudi Arabia	1 (1.9)
	Singapore	1 (1.9)
	Thailand	1 (1.9)
	Australia	1 (1.9)
	Canada	1 (1.9)
	Egypt	1 (1.9)
	Nigeria	1 (1.9)
	South Africa	1 (1.9)
**Publication type**		
	Conference proceedings	31 (58.5)
	Journals	22 (41.5)

**Figure 4:**
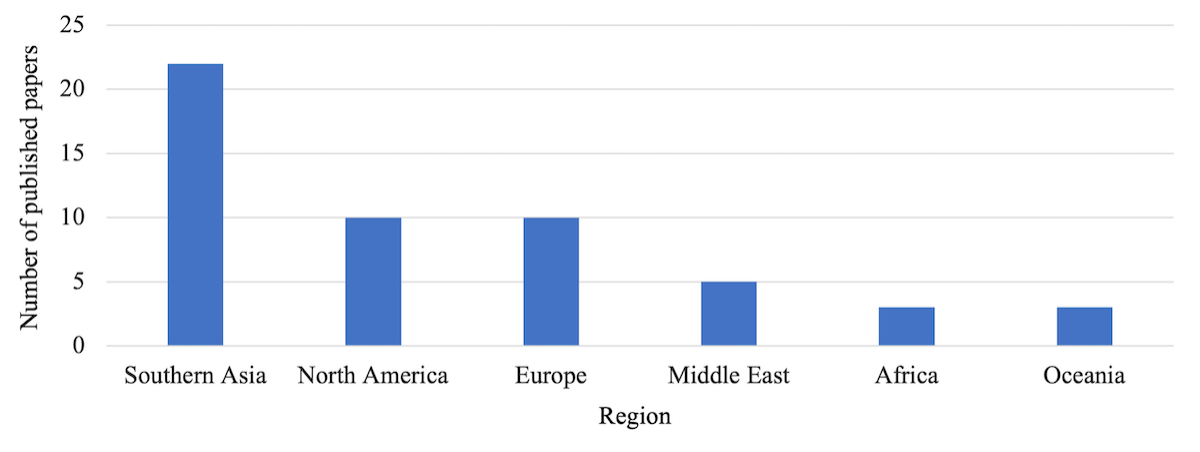
Number of published papers by region.

Figure 4 shows the region of publication of the included studies. The studies included were published in different parts of the world. In Southern Asia, 22 studies (41.5%) were conducted in China, India, Bangladesh, Indonesia, Pakistan, Singapore, South Korea, and Thailand; 10 studies (18.9%) were conducted in North America, specifically the United States and Canada; 10 studies were conducted in Europe, including Austria, Poland, Germany, France, the United Kingdom, and Russia; 5 studies (9.4%) were conducted in the Middle East, including Lebanon, Turkey, Iran, and Saudi Arabia; 3 studies (5.7%) were conducted in Africa, specifically Egypt, South Africa, and Nigeria; and in Oceania, 3 studies were concluded in New Zealand and Australia.

The selected studies were either published in conference proceedings or journals: 31 of 53 studies (58.5%) were published in conference proceedings, and the rest of the papers (22/53, 41.5%) were published in journals. Multimedia Appendix 3 displays the characteristics of each included study.

### Data Characteristics

Table 2 summarizes the characteristics of the used data in the selected studies. The studies used different sizes of data sets to train their models. The average number of used images in the selected studies was around 7800. The lowest number of images used was 40 [24], whereas the highest number of images used was 129,450 [23]. We categorized these data set sizes into three groups, depending on the number of images used. The first category contained small data sets that had fewer than 1000 images (21/53, 39.6%). The second category used medium-size data sets consisting of 1000-10,000 images (25/53, 47.2%). The last category contained large data sets that included more than 10,000 images (7/53, 13.2%).

**Figure 5:**
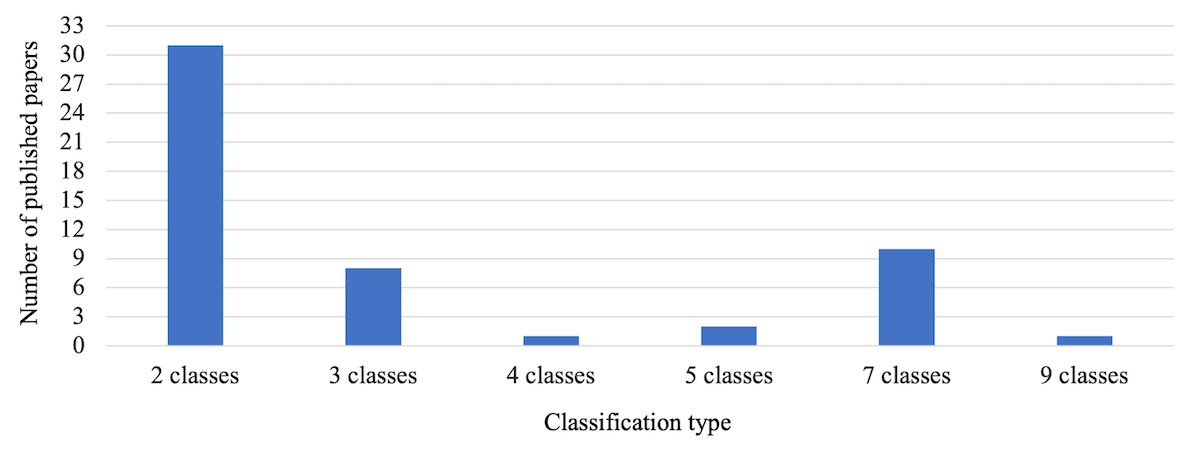
Number of published papers by number of diagnostic classes used.

We divided the papers into two groups based on the classification type. We found that more than half of the papers (31/53, 58.5%) built models to classify whether the lesion was benign or malignant (two-class/binary classification). The rest of the papers (22/53, 41.5%) presented models in which skin lesions were classified using three or more diagnostic classes (multiclass classification). Figure 5 shows the number of papers using different diagnostic classes. In the multiclass classification, 8 studies used 3 diagnostic classes, 1 study used 4 classes, 2 studies used 5 classes, 10 studies used 7 classes, and 1 study used 9 classes. The benign classes included benign keratosis, melanocytic nevus, and dermatofibroma. The malignant classes included melanoma and basal cell carcinoma. Other lesions, such as vascular lesions, actinic keratosis, genodermatosis, and tumors, could be either benign or malignant.

**Table 2:** Data and deployment characteristics (N=53).

Characteristics		n (%)
**Data set size**		
	Small	21 (39.6)
	Medium	25 (47.1)
	Large	7 (13.2)
**Classification type**		
	2 classes	31 (58.5)
	3 classes	8 (15.1)
	4 classes	1 (1.9)
	5 classes	2 (3.8)
	7 classes	10 (18.9)
	9 classes	1 (1.9)
**Image type**		
	Dermoscopic	43 (81.1)
	Clinical	5 (9.4)
	High quality	4 (7.5)
	Spectroscopic	1 (1.9)
**Deployment**		
	Development	45 (84.9)
	System	3 (5.7)
	Web application	3 (5.7)
	Mobile application	2 (3.8)

With regard to the type of images used to train, test, and validate the models, 43 of 53 studies (81.1%) used dermoscopic images; 5 studies (9.4%) used clinical images that were taken using a normal camera; and 4 studies (7.5%) used high-quality images that were taken with a professional camera. The remaining study used spectroscopic images requiring a specialized system taking images of a lesion from three different spots using polarized and unpolarized light.

The majority of the studies (45/53, 84.9%) presented technologies that are still in the development phase. The rest of the studies (8/53, 15.1%) have been deployed into a usable form: 3 studies developed a health care system, 3 studies deployed the model into a mobile application, and 2 studies transferred the model into a web application. Multimedia Appendix 4 displays the data and deployment characteristics of each included study.

### Diagnostic Techniques

We categorized the papers into two groups based on the AI technique used in detecting and classifying skin cancer. The groups were *shallow* techniques and *deep* techniques. These two groups differed mainly in the complexity of the AI architecture underlying the model. *Shallow* techniques use either simple machine learning algorithms, such as a support vector machine (SVM), or only a couple of layers of neural networks [63]. If, in contrast, the AI architecture is a neural network that consists of at least three layers, it is categorized as a *deep* technique [19]. It turns out that around a quarter of the studies (14/53, 26.4%) used shallow techniques, while the rest (39/53, 73.6%) used deep techniques. Within each of the groups, studies may have used different models or algorithms, and some studies proposed multiple methods or provided testing data using multiple methods. In this study, we only considered the model that had the best-reported performance in each paper.

As shown in Table 3, most studies that used *shallow* techniques adopted an SVM (9/14, 64.3%), which is a common two-class classifier that uses a hyperplane as a decision boundary [6]. The rest of the studies (5/14, 35.7%) adopted the naive Bayes (NB) algorithm (1/14, 7.1%), which is a probabilistic classifier that assumes conditional independence among the features [6]; logistic regression (LR; 1/14), which uses probability for prediction; k-nearest neighbors (kNNs; 1/14), which classify a sample based on samples close to it; and random forests (RFs; 1/14), which classify using decision trees [6]. A hybrid model (1/14) classified images through multiple iteratives using Adaboost and an SVM.

**Table 3:** Techniques used in included studies using shallow techniques (N=14).

Model	n (%)	Reference
SVM^a^	9 (64.3)	[12,15,16,19,21,26,27,29,60]
NB^b^	1 (7.1)	[11]
LR^c^	1 (7.1)	[13]
kNN^d^	1 (7.1)	[25]
RF^e^	1 (7.1)	[28]
Hybrid	1 (7.1)	[18]

^a^SVM: support vector machine.

^b^NB: naive Bayes.

^c^LR: logistic regression.

^d^kNN: k-nearest neighbor.

^e^RF: random forest.

The majority of the studies that used *deep* techniques (Table 4) adopted different types of convolutional neural networks (CNNs; 36/39, 92.3%), which assign importance to parts of images using ImageNet-pretrained architectures (18/39, 46.2%), including the residual network (ResNet), Inception, AlexNet, MobileNet, Visual Geometry Group (VGG), Xception, DenseNet, and GoogleNet. In addition, some of the CNN-based studies (11/39, 28.2%) built customized CNNs or ResNets. Moreover, some studies adopted different combinations of CNNs along with other models (hybrid models; 5/39, 12.8%), as well as using ensemble models (4/39, 10.3%); the remaining study (1/39, 2.6%) used the OpenCV library. Multimedia Appendix 5 provides further details regarding each of the models in terms of the method used, the number of layers (ranging from 1 to 121 layers), the method used for selecting the hyperparameters, and the performance of the proposed model with respect to other reported models within the study.

**Table 4:** Techniques used in included studies using deep techniques (N=39).

Model		n (%)	Reference
**Pretrained CNNs^a^**			
	ResNet^b^	5 (12.8)	[22,41,49,50,54]
	Inception	3 (7.7)	[23,42,56]
	AlexNet	3 (7.7)	[34,35,39]
	MobileNet	3 (7.7)	[45,51,55]
	VGG^c^	2 (5.1)	[30,52]
	Xception	1 (2.6)	[43]
	DenseNet	1 (2.6)	[58]
**Custom**			
	CNN	9 (23.1)	[14,24,40,47,53,57,59,61,62]
	ResNet	2 (5.1)	[31,33]
Hybrid		5 (12.8)	[17,32,38,44,46]
Ensemble		4 (10.3)	[20,36,37,48]
OpenCV		1 (2.6)	[10]

^a^CNN: convolutional neural network.

^b^ResNet: residual network.

^c^VGG: Visual Geometry Group.

### Evaluation Metrics

The studies included in this scoping review used different evaluation metrics to assess their proposed models. In the studies, the following five primary evaluation metrics were used to assess the built models: accuracy, sensitivity and specificity, positive predictive value (PPV) or precision, area under the curve (AUC), and F1-score. All five metrics ranged from 0% to 100%; the higher the score, the better the model performance. To compute the different evaluation metrics, the following types of samples were identified: First, true positives (TPs), which are malignant samples that the AI tool also detected as malignant; second, false positives (FPs), which are benign samples that the AI tool detected as malignant; third, true negatives (TNs), which are benign samples that were also detected as benign by the AI tool; and fourth, false negatives (FNs), which are malignant samples that were detected as benign by the AI tool. It is worth mentioning that more than half of the studies (33/53, 62.3%) reported multiple evaluation metrics, in addition to the primary metric.

Accuracy = (TP + TN)/(TP + TN + FP + FN), which implies how well the model detects the diagnostic classes, was reported in the majority of the papers (44/53, 83%). Sensitivity or recall = TP/(TP + FN), which is the probability of the model, given only malignant samples, to correctly diagnose them as malignant, was reported in 30 (56.6%) papers. Specificity = TN/(TN + FP), which determines the proportion of negative samples that are correctly detected, was reported in 24 (45.3%) papers. The PPV or precision = TP/(TP + FP) was reported in 13 (24.5%) papers. The AUC, which is the area of the receiver operating characteristic (ROC) curve and plots the TP against the FP, was reported in 11 (20.8%) papers. The F1-score, which is the harmonic mean of recall and precision, was reported in 9 (16.9%) papers. In addition, the dice coefficient = 4TP/(FN + 2TP + FP) was reported in 4 (7.5%) papers. The negative predictive value (NPV) = TN/(TN + FN) was reported in 2 (3.8%) papers. The Jaccard index = 2TP/(TP + FN + FP) was reported in 2 papers. The Cohen κ was also reported in 2 papers. Finally, the Youden index = sensitivity + specificity – 1 was reported in 1 (1.9%) paper.

Herein, we conducted our analysis of each paper based on the best-performing experiment in case multiple experiments were conducted. In addition, if multiple evaluation metrics were used, we used the primary evaluation metric score that was reported by the authors in the abstract or conclusion as the main focus of the paper or the used average score of each of the diagnostic classes for multiclass classification papers. Of the aforementioned metrics, accuracy, AUC, sensitivity and specificity, and the F1-score were used as the primary evaluation metrics. Around 73% (39/53) of the papers used accuracy as their primary evaluation metric to assess the trained models. The average accuracy value was 86.8%, with a maximum of 98.8% [60] and a minimum of 67% [10]. The AUC was reported in 9 studies, with an average score of 87.2%; the highest AUC score was 91.7% [41], whereas the lowest AUC score was 82.0% [26]. Sensitivity and specificity were used in 4 studies, and the F1-score was reported in 1 study. Multimedia Appendix 6 shows the data characteristics, used model, and evaluation scores for each included study (Table 5).

**Table 5:** Primary evaluation metrics and scores reported by included studies (N=53).

Score		Reference
**Accuracy**		
	99%	[60]
	98%	[21,27]
	96%	[24]
	95%	[17,22,61]
	94%	[20,40]
	93%	[16]
	92%	[18]
	91%	[51,52,62]
	90%	[36,42,57]
	89%	[11,43]
	88%	[13,48]
	87%	[25,49,53]
	86%	[35,44,58]
	84%	[34]
	83%	[54,55]
	81%	[14]
	80%	[19]
	77%	[28]
	75%	[39,47,59]
	72%	[23,56]
	67%	[10]
**AUC^a^**		
	92%	[41]
	91%	[33,38]
	89%	[32]
	87%	[46]
	85%	[37,50]
	84%	[30]
	82%	[26]
**Sensitivity**		
	96%	[31]
	90%	[15]
	83%	[12]
	77%	[29]
**Specificity**		
	96%	[15]
	90%	[12]
	89%	[31]
	70%	[29]
**F1-score**		
	83%	[45]

^a^AUC: area under the curve.

## Discussion

### Main Findings

We studied multiple characteristic types for the 53 selected studies. First, we included the study characteristics. Most studies were published in 2019, the majority of the studies were published in Southern Asia, and most studies were published in journals. Second, we discussed the data characteristics. For training and testing, most of the studies used medium-size data sets, the majority of the studies built binary classifiers, and dermoscopic images were used the most. Third, we categorized the adopted AI models into shallow and deep. Most shallow models were SVM based, whereas most deep models were CNN-based neural networks. Generally, deep models were adopted more than shallow models. Fourth, we listed the evaluation metrics used along with the reported scores to assess the performance of the models. In total, 11 different evaluation metrics were used, where accuracy was the most commonly used metric, so we focused on accuracy.

### Performance Factors

**Figure 6:**
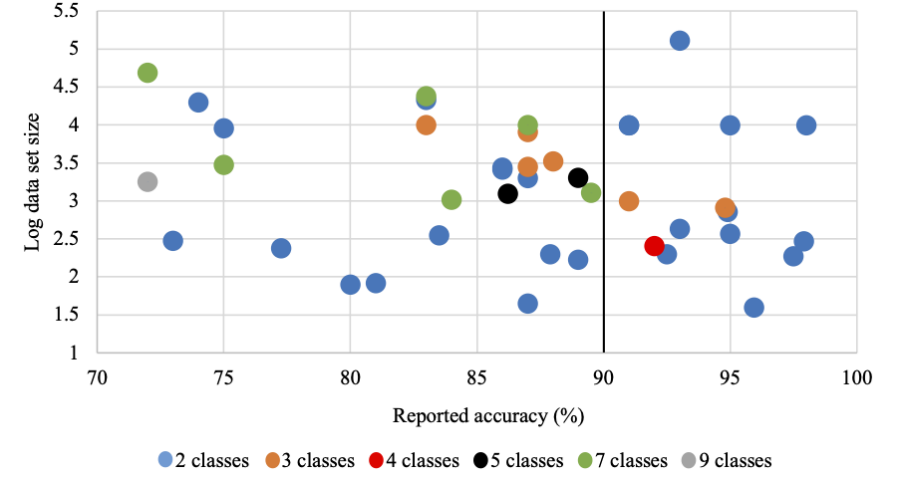
Effect of the number of diagnostic classes and data set size on accuracy.

After analyzing the reported performance scores, we concluded that there is a correlation between the performance and the number of classes used. In addition, another factor that affects the performance is the data set size. Next, we study this hypothesis with respect to accuracy since most of the studies (39/53, 73.6%) used it as the primary evaluation metric, although it might not be the most fitted evaluation metric to assess such a task, especially in the case of imbalanced data. We believe that having a confusion matrix or the number of TPs, FPs, TNs, and FNs would avoid bias and give a clearer evaluation of how the model behaves with regard to each of the diagnostic classes. From the studies, the top accuracy scores were ~98% [21,27,60]. In studies leading to this accuracy, the authors built a two-class classification (benign vs malignant) model using data sets of 200, 356, and 200 images, respectively. The top 10 accuracy scores (99%-92%) also built two-class classifiers using an average of around 800 images. In addition, 26 studies built two-class classifiers with an average accuracy score of around 88% using an average data set size of around 1000 images, while 17 studies built multiclass classifiers with an average accuracy score of 85%; they used around 15,000 images on average. The second-lowest accuracy score was 72% [23], in which the authors developed a multiclass classifier using 9 different diagnostic classes and 129,450 images, which is the highest number of classes and the biggest data set size included in this study. Figure 6 plots the logarithmic data set size over accuracy, using colors to indicate the number of diagnostic classes. As can be seen, accuracy increases as the number of diagnostic classes and data set size decreases. Specifically, after the threshold of 90% in accuracy, we can see that the majority of the studies built two-class classifiers. The factors that might be behind such a pattern are further discussed next.

### Classification Type Factor

Binary classifiers tend to have better performance when compared to multiclass classifiers. This seems intuitively right since binary classifiers are less expressive. Instead of distinguishing between several classes, binary classifiers have “less to learn.” To illustrate this point, let us compare limits on the probability of each class for a binary and a five-class classifier. For the five-class classifier, there must be at least one class with a probability of ≤20% (according to the *pigeonhole principle* [64]). Predicting this low probability class is, therefore, typically harder than in the case of a binary classifier, for which we know that there exists exactly (and, thus, at most) one class with a probability of ≤50%. Another way of looking at it is to consider an algorithm that performs a random choice assuming perfectly balanced data. In the binary case, the error rate of this algorithm would be 50%, whereas for the five-class classifier, it increases to 80%, a 1.6-fold increase. The problem may be further exacerbated by imbalanced data, which often arises naturally due to differences in the prevalence rates of medical conditions. Therefore, it is also not surprising that binary classifiers work well, given less data for training, since the model may still be fed sufficient numbers of examples for each class.

### Data Set Size Factor

However, what is surprising is that Figure 6 suggests that the performance increased with decreasing training data. To this end, we would like to note that the two methods with the best performance used shallow techniques that tend to be far less hungry for data than deep methods, since manual feature engineering is often part of the pipeline. Furthermore, Afifi et al [21] used clinical image data, which may be of superior quality. In addition, depending on the testing setup, it cannot be ruled out that methods relying on less data lack the generality of models that have been trained using large volumes of data. In such scenarios, the models would be closer to data retrieval machines due to overfitting than general detectors and classifiers. To fully assess apparent issues such as this, it is important not to rely on a single performance metric when reporting results. Especially, sensitivity and specificity can be as important as accuracy in this context since they model FN and FP rates. All considered, we would, therefore, like to reiterate our earlier statement that we believe it is important for any AI to undergo rigorous clinical studies and testing before being deployed in a clinical environment.

### Technique Type Factor

With regard to the techniques described in the studies included in this review, deep and shallow models (regardless of the number of layers) have similar performances. For example, within the shallow models, the top five skin cancer detectors were built using an SVM with accuracy scores of 93%-99% using relatively small data sets. The SVM was the most commonly used method among the shallow models. Similarly, within the deep models, the top five CNN-based skin cancer detectors had 94%-96% accuracy using medium-size data sets. CNNs were also the most commonly used method among the deep models. Theoretically, deep neural networks tend to have better performance with regard to image classifications [65]. One reason is that shallow models are often limited to less expressive functional spaces when compared to deep networks. From a technical perspective, this may well explain their lower performance due to a lack of the ability to fully capture the complex nature of images during training. In contrast, deep networks and CNNs can learn features at multiple scales and complexity to provide fast diagnoses [66]. Therefore, they not only detect, select, and extract features from medical images but also contribute by enhancing and constructing new features from the medical images [67]. Such similarities and inconsistencies in the performances of the included studies are due to the diverse evaluation metrics used, the data set size, image types, and the number of diagnostic classes among the studies.

### Publication Year

Based on the study characteristics, we noticed that the number of published papers has increased since 2016 and that most papers discuss the use of dermoscopic images, making it the most used image modality for the detection and classification of skin cancer. We believe that this is because the International Skin Imaging Collaboration (ISIC) competition started in 2016 [8], which offered several medical data sets of dermoscopic images that have ever since been used to build AI-based models. Most of these studies are still in the development stage, and we firmly believe that these models still need to be further validated and tested in hospitals. However, dermatologists and patients are beginning to adapt to the notion of relying on AI to diagnose skin cancer.

### Practical and Research Implications

In this scoping review, we summarized the findings in the literature related to diagnosing skin cancer by using AI-based technology. We also categorized the papers included in this review based on the methodology used, the type of AI techniques, and their performance, and found the link between these aspects.

We noted that although all the papers included in this scoping review discuss the application and performance of a specific AI technology, the reporting is performed heterogeneously. A discussion of the relationship between using one specific AI technique and other aspects, such as data set size, or even a discussion of why the evaluation metric used is reasonable is normally not attempted. This, of course, potentially hampers research in this direction, as it becomes harder for future studies to provide a comprehensive comparison with the existing work that follows scientific rigor. This scoping review filled this gap by performing the necessary characterizations and analyses. This was achieved by grouping each of the used AI technologies into shallow and deep approaches, linking each type to the evaluation metrics used, listing and interpreting the number of diagnostic classes used in each study, and highlighting the dependency of performance on data set size and other factors. To the best of our knowledge, no similar work has been performed to fill this gap. In the Conclusion section, we will highlight our main findings.

### Limitations

This scoping review examined papers that were published between January 2009 and July 2020, and any published study outside this time line was excluded, which may have excluded older AI-based methods. In addition, we examined papers written in English; other languages were not included, which may have led to the exclusion of some studies conducted in other parts of the world. Another limitation might be the gap between the time the research was performed and the time the work was submitted, which excluded published papers during that period. Although we applied all due diligence, a small residual chance of accidentally having overlooked papers in an academic database cannot be fully ruled out. In addition, although we tried to discuss all findings in the literature, it is beyond the scope of this review to detail every single finding of the papers. Similarly, an investigation into data biases in the literature (imbalanced data with respect to diagnostic classes, patient ethnicity and skin color, gender, etc) is left as a direction for future studies.

### Conclusions

The use of AI has high potential to facilitate the way skin cancer is diagnosed. Two main branches of AI are used to detect and classify skin cancer, namely shallow and deep techniques. However, the reliability of such AI tools is questionable since different data set sizes, image types, and number of diagnostic classes are being used and evaluated with different evaluation metrics. Accuracy is the metric used most as a primary evaluation metric but does not allow for independently assessing FN and FP rates. This study found that higher accuracy scores are reported when fewer diagnostic classes are included. Interestingly and counterintuitively, our analysis also suggests that higher accuracy scores are reported when smaller sample sizes are included, which may be due to factors such as the type of images and the techniques used. Furthermore, only independent, external validation using a large, diverse, and unbiased database is fit to demonstrate the generality and reliability of any AI technology prior to clinical deployment.

## Appendix

Multimedia Appendix 1 Search query.

Multimedia Appendix 2 Data extraction form.

Multimedia Appendix 3 Study characteristics.

Multimedia Appendix 4 Data and deployment characteristics.

Multimedia Appendix 5 Technical details.

Multimedia Appendix 6 Data, model, and evaluation.

## Abbreviations

ACM DL: Association for Computing Machinery Digital Library
AI: artificial intelligence
AUC: area under the curve
CNN: convolutional neural network
FN: false negative
FP: false positive
IEEE: Institute of Electrical and Electronics Engineers
ISIC: International Skin Imaging Collaboration
kNN: k-nearest neighbor
LR: logistic regression
NB: naive Bayes
NPV: negative predictive value
PPV: positive predictive value
PRISMA-ScR: Preferred Reporting Items for Systematic Reviews and Meta-Analyses Extension for Scoping Reviews
ResNet: residual network
RF: random forest
ROC: receiver operating characteristic
SVM: support vector machine
TN: true negative
TP: true positive
VGG: Visual Geometry Group
