# Supplementary material for: Artificial Intelligence for Skin Cancer Detection: Scoping Review
Source: J Med Internet Res. 2021 Nov 24;23(11):e22934. doi: 10.2196/22934 (PMC8663507; doi:10.2196/22934)
Supplement: Multimedia Appendix 1 [file jmir_v23i11e22934_app1.docx]

## Multimedia Appendix 1: Search query

Database(s): **Ovid MEDLINE(R)**1946 to June Week 4 2020
Search Strategy:

| **#** | **Searches** | **Results** |
| --- | --- | --- |
| 1 | exp Skin Neoplasms/ | 124759 |
| 2 | skin cancer*.tw. | 18674 |
| 3 | Skin Neoplasm*.tw. | 588 |
| 4 | melanoma.tw. | 98067 |
| 5 | skin tumor*.tw. | 5800 |
| 6 | skin lesion*.tw. | 25222 |
| 7 | basal cell carcinoma.tw. | 9847 |
| 8 | cutaneous B-cell lymphoma.tw. | 401 |
| 9 | cutaneous T-cell lymphoma.tw. | 3621 |
| 10 | sebaceous carcinoma.tw. | 628 |
| 11 | squamous cell carcinoma.tw. | 72999 |
| 12 | exp Artificial Intelligence/ | 96910 |
| 13 | artificial intelligence.tw. | 3847 |
| 14 | exp Machine Learning/ | 18494 |
| 15 | Machine learning.tw. | 15939 |
| 16 | exp Deep Learning/ | 2322 |
| 17 | Deep learning.tw. | 3537 |
| 18 | Neural network*.tw. | 31848 |
| 19 | Diagnosis/ | 17338 |
| 20 | Diagnos*.tw. | 2114381 |
| 21 | screen*.tw. | 635364 |
| 22 | detect*.tw. | 2006377 |
| 23 | classif*.tw. | 521487 |
| 24 | 1 or 2 or 3 or 4 or 5 or 6 or 7 or 8 or 9 or 10 or 11 | 281828 |
| 25 | 12 or 13 or 14 or 15 or 16 or 17 or 18 | 114773 |
| 26 | 19 or 20 or 21 or 22 or 23 | 4534001 |
| 27 | 24 and 25 and 26 | 519 |
| 28 | limit 27 to yr="2009 -Current" | 398 |
| 29 | limit 28 to English language | 390 |

| Database | Query | Hits |
| --- | --- | --- |
| IEEE Xplore | (("skin neoplasm*" OR "skin cancer*" OR "melanoma" OR "skin tumor*" OR "skin lesion*" OR "basal cell carcinoma" OR "cutaneous B-cell lymphoma" OR "cutaneous T-cell lymphoma" OR "sebaceous carcinoma" OR "squamous cell carcinoma") AND ("artificial intelligence" OR "machine learning" OR "deep learning" OR "neural network*") AND ("diagnos*" OR "screen*" OR "detect*" OR "classif*")) | 384 |
| ACM Digital library | (("skin neoplasm*" OR "skin cancer*" OR "melanoma" OR "skin tumor*" OR "skin lesion*" OR "basal cell carcinoma" OR "cutaneous B-cell lymphoma" OR "cutaneous T-cell lymphoma" OR "sebaceous carcinoma" OR "squamous cell carcinoma") AND ("artificial intelligence" OR "machine learning" OR "deep learning" OR "neural network*") AND ("diagnos*" OR "screen*" OR "detect*" OR "classif*")) | 124 |
