# Supplementary material for: Artificial Intelligence for Skin Cancer Detection: Scoping Review
Source: J Med Internet Res. 2021 Nov 24;23(11):e22934. doi: 10.2196/22934 (PMC8663507; doi:10.2196/22934)
Supplement: Multimedia Appendix 2 [file jmir_v23i11e22934_app2.docx]

## Multimedia Appendix 2 – Data extraction form

| **Concept** | **Definition** |
| --- | --- |
| **Study characteristics** |  |
| Author | The first author of the study. |
| Year of Publication | The year in which the study was published. |
| Country of publication | The country where the study was published. |
| Type of publication | The medium in which the study was published (e.g., journal article, conference proceedings, dissertation). |
| Study type | The type of the research conducted (e.g. experiments, comparisons, review, evaluation). |
| Study aim | What the study aimed to find out. |
| **Data characteristics** |  |
| Dataset size | The number of images used to train and test the model. |
| Classification type | The type and number of diagnostic classes used (binary classification, multi-class classification (3 classes, 4 classes, 5 classes, … etc.)). |
| Image type | The type of images used to train and test the model (e.g. dermoscopic, clinical, high quality). |
| **Deployment stage** | The form of the final product of the proposed model (e.g. under development, mobile application, web application). |
| Used environment | The form of the final product of the proposed model (e.g. under development, mobile application, web application). |
| **Diagnostic Techniques** |  |
| Type of Al | The type of the used AI-based technique (e.g. shallow machine learning technique, deep neural networks). |
| AI algorithm | The name of the used AI-based algorithm (e.g. support vector machine, Random forest, K-nearest neighbor, Convolutional Neural Networks). |
| Type of neural network | The type of the learning used (e.g. built from scratch, pre-trained model, hybrid). |
| Name of pre-trained model | The name of the pre-trained architecture (e.g. ResNet, AlexNet, Inception, Xception). |
| **Technical evaluation** |  |
| Evaluation metrics | The primary used evaluation metric to assess the proposed model (e.g. accuracy, specificity, Area Under Receiver Operating Characteristic Curve, F1-Score). |
| Evaluation score | The score of the assessment based on the evaluation metric (out of 100%). |
