# Supplementary material for: Artificial Intelligence for Skin Cancer Detection: Scoping Review
Source: J Med Internet Res. 2021 Nov 24;23(11):e22934. doi: 10.2196/22934 (PMC8663507; doi:10.2196/22934)
Supplement: Multimedia Appendix 3 [file jmir_v23i11e22934_app3.docx]

## Multimedia Appendix 3: Study characteristics

| **Ref** | **Year** | **Type** | **Country** |
| --- | --- | --- | --- |
| [10] | 2011 | Conference | USA |
| [11] | 2014 | Journal | USA |
| [12] | 2014 | Conference | New Zealand |
| [13] | 2015 | Journal | USA |
| [14] | 2016 | Conference | Iran |
| [15] | 2016 | Journal | Poland |
| [16] | 2016 | Journal | Poland |
| [17] | 2016 | Conference | Australia |
| [18] | 2016 | Journal | India |
| [19] | 2017 | Conference | Nigeria |
| [20] | 2017 | Journal | China |
| [21] | 2017 | Conference | New Zealand |
| [22] | 2017 | Journal | Hong Kong |
| [23] | 2017 | Journal | USA |
| [24] | 2018 | Conference | France |
| [25] | 2018 | Conference | Philippines |
| [26] | 2018 | Journal | USA |
| [27] | 2018 | Journal | Pakistan |
| [28] | 2018 | Journal | India |
| [29] | 2018 | Journal | Lebanon |
| [30] | 2018 | Journal | Korea |
| [31] | 2018 | Conference | USA |
| [32] | 2018 | Conference | Singapore |
| [33] | 2018 | Journal | China |
| [34] | 2018 | Conference | Turkey |
| [35] | 2018 | Conference | UK |
| [36] | 2018 | Conference | Egypt |
| [37] | 2018 | Conference | USA |
| [38] | 2018 | Conference | Austria |
| [39] | 2018 | Conference | USA |
| [40] | 2019 | Journal | Pakistan |
| [41] | 2019 | Journal | China |
| [42] | 2019 | Conference | Turkey |
| [43] | 2019 | Conference | Russia |
| [44] | 2019 | Conference | India |
| [45] | 2019 | Conference | China |
| [46] | 2019 | Conference | China |
| [47] | 2019 | Conference | USA |
| [48] | 2019 | Journal | Austria |
| [49] | 2019 | Conference | Poland |
| [50] | 2019 | Journal | Germany |
| [51] | 2019 | Conference | Saudi Arabia |
| [52] | 2019 | Conference | Bangladesh |
| [53] | 2019 | Conference | Canada |
| [54] | 2019 | Conference | Indonesia |
| [55] | 2019 | Conference | Thailand |
| [56] | 2019 | Conference | Indonesia |
| [57] | 2019 | Conference | Bangladesh |
| [58] | 2020 | Journal | China |
| [59] | 2020 | Journal | Germany |
| [60] | 2020 | Journal | India |
| [61] | 2020 | Journal | South Africa |
| [62] | 2020 | Conference | India |
