# Supplementary material for: Artificial Intelligence for Skin Cancer Detection: Scoping Review
Source: J Med Internet Res. 2021 Nov 24;23(11):e22934. doi: 10.2196/22934 (PMC8663507; doi:10.2196/22934)
Supplement: Multimedia Appendix 4 [file jmir_v23i11e22934_app4.docx]

| **Ref** | **Dataset size** | **Classification** | **Image Type** | **Deployment Stage** |
| --- | --- | --- | --- | --- |
| [24] | 40 | 2 classes | High quality | Development |
| [25] | 45 | 2 classes | Dermoscopic | Healthcare system |
| [19] | 80 | 2 classes | High quality | Development |
| [10] | 83 | 2 classes | High quality | Mobile application |
| [26] | 100 | 2 classes | Dermoscopic | Development |
| [14] | 170 | 2 classes | Clinical | Development |
| [11] | 187 | 2 classes | Spectroscopic | Healthcare system |
| [15] | 200 | 2 classes | Dermoscopic | Development |
| [27] | 200 | 2 classes | Dermoscopic | Development |
| [60] | 200 | 2 classes | Dermoscopic | Development |
| [20] | 240 | 2 classes | Dermoscopic | Development |
| [13] | 256 | 2 classes | Dermoscopic | Development |
| [28] | 294 | 2 classes | Clinical | Development |
| [21] | 356 | 2 classes | Clinical | Development |
| [29] | 369 | 2 classes | Dermoscopic | Development |
| [12] | 370 | 2 classes | Clinical | Development |
| [30] | 724 | 2 classes | Dermoscopic | Development |
| [17] | 814 | 2 classes | Dermoscopic | Development |
| [18] | 992 | 2 classes | Dermoscopic | Development |
| [58] | 1,031 | 2 classes | Dermoscopic | Development |
| [22] | 1,250 | 2 classes | Dermoscopic | Development |
| [40] | 1,276 | 2 classes | Dermoscopy | Development |
| [31] | 1,520 | 2 classes | Dermoscopic | Development |
| [59] | 1,796 | 2 classes | Dermoscopic | Healthcare system |
| [61] | 2,600 | 2 classes | Dermoscopic | Development |
| [42] | 3,297 | 2 classes | Dermoscopic | Development |
| [39] | 13,025 | 2 classes | High quality | Development |
| [50] | 14,016 | 2 classes | Dermoscopic | Development |
| [54] | 21,659 | 2 classes | Dermoscopic | Development |
| [57] | 23,907 | 2 classes | Dermoscopic | Development |
| [51] | 48,373 | 2 classes | Dermoscopic | Mobile application |
| [49] | 429 | 3 classes | Dermoscopic | Development |
| [32] | 2,000 | 3 classes | Dermoscopic | Development |
| [33] | 2,000 | 3 classes | Dermoscopic | Development |
| [37] | 2,000 | 3 classes | Dermoscopic | Development |
| [41] | 2,000 | 3 classes | Dermoscopy | Development |
| [46] | 2,750 | 3 classes | Dermoscopic | Development |
| [48] | 2,787 | 3 classes | Dermoscopic | Development |
| [44] | 3,000 | 3 classes | Dermoscopic | Development |
| [16] | 300 | 4 classes | Dermoscopic | Development |
| [35] | 9,144 | 5 classes | Clinical | Development |
| [43] | 20,000 | 5 classes | Dermoscopic | Web application |
| [34] | 8,011 | 7 classes | Dermoscopic | Development |
| [36] | 10,000 | 7 classes | Dermoscopy | Development |
| [38] | 10,000 | 7 classes | Dermoscopic | Development |
| [45] | 10,000 | 7 classes | Dermoscopic | Development |
| [47] | 10,000 | 7 classes | Dermoscopic | Mobile application |
| [52] | 10,000 | 7 classes | Dermoscopic | Development |
| [55] | 10,000 | 7 classes | Dermoscopic | Development |
| [56] | 10,000 | 7 classes | Dermoscopic | Web application |
| [62] | 10,000 | 7 classes | Dermoscopic | Development |
| [53] | 10,000 | 7 classes | Dermoscopic | Development |
| [23] | 129,450 | 9 classes | Dermoscopic | Development |

## Multimedia Appendix 4: Data and deployment characteristics
