# Supplementary material for: Artificial Intelligence for Skin Cancer Detection: Scoping Review
Source: J Med Internet Res. 2021 Nov 24;23(11):e22934. doi: 10.2196/22934 (PMC8663507; doi:10.2196/22934)
Supplement: Multimedia Appendix 5 [file jmir_v23i11e22934_app5.docx]

## **Multimedia Appendix 5: Technical Details**

| **Ref** | **Shallow Model** | **Method** | **Parameters** | **Performance Comparisons** | **Outperformed?** |
| --- | --- | --- | --- | --- | --- |
| [12] | SVM | Cascading two SVMs | Predefined | Other shallow models | Yes |
| [15] | SVM | SVM-based classification | Predefined | - | - |
| [16] | SVM | SVM with radial basis function kernel | Tuned | Other shallow and deep models | Yes |
| [19] | SVM | SVM-based classification | Tuned | - | - |
| [21] | SVM | 2-stage cascaded classification | Tuned | Multiple experiments | - |
| [26] | SVM | Non-Linear SVM Fusion | - | Other shallow models | Yes |
| [27] | SVM | SVM-based classification | Tuned | Models in literature | Yes |
| [29] | SVM | SVM-based classification | Tuned | Other shallow and deep models | Yes |
| [60] | SVM | Using bendlet transform features | Optimized | Models in literature | Yes |
| [11] | NB | Using numeric estimator classes | Default | Other shallow and deep models | Yes |
| [13] | LR | Multivariate analysis | Optimized | Multiple experiments | - |
| [25] | KNN | Classification of geometrical features | - | - | - |
| [28] | RF | - | Tuned | Other shallow models | Yes |
| [18] | Hybrid | Hybrid AdaBoost and SVM model | Predefined | Other shallow and deep models | Yes |
| SVM: support vector machine, NB: Naïve Bayes, LR: logistic regression, KNN: K-nearest neighbors, “-”: item was not discussed in the paper. | | | | | |

| **Ref** | **Deep Model** | **Method** | **No. of Layers** | **Parameters** | **Performance Comparisons** | **Outperformed?** |
| --- | --- | --- | --- | --- | --- | --- |
| **Pretrained CNN-based models** | | | | | | |
| [22] | ResNet | Fully convolutional residual network | 50 | - | Different depths of ResNets | Yes |
| [41] | ResNet | - Attention residual learning CNN  - Multiple kernel sizes | 50 | Tuned | Different depths of ResNets | Yes |
| [49] | ResNet | Kernel size: 3 × 3 | - | Tuned | - | - |
| [50] | ResNet | ResNet-50 | 50 | Tuned | Top 5 ranks of ISBI | Yes |
| [54] | ResNet | ResNet-50 | 50 | Predefined | Different depths of ResNets | Yes |
| [23] | Inception | GoogleNet Inception v3 | 48 | Tuned | - | - |
| [42] | Inception | GoogleNet Inception v3 | 48 | - | Different depths of ResNets | Yes |
| [56] | Inception | GoogleNet Inception v3 | 48 | Predefined | MobileNet V1 | Yes |
| [34] | AlexNet | - | 19 | Predefined | - | - |
| [35] | AlexNet | Linear error-correcting output codes | 8 | - | Other shallow and deep models. | No |
| [39] | AlexNet | - | 8 | Tuned | Different hyperparameters of AlexNet | Yes |
| [45] | MobileNet | - | 28 | Tuned | - | - |
| [51] | MobileNet | - | 28 | Predefined | - | - |
| [55] | MobileNet | - | 28 | Optimized | Different hyperparameters of MobileNet | - |
| [30] | VGG | - VGG-16  - Kernel size: 3 × 3  - Feature maps: 64 | 16 | Tuned | Different CNNs | Yes |
| [52] | VGG | - VGG-16  - Kernel size: 2 × 2 | 16 | Default | Different CNNs | No |
| [43] | Xception | - | 71 | Tuned | Different CNNs | Yes |
| [58] | DenseNet | DenseNet-121 | 121 | Predefined | Different CNNs | Yes |
| [14] |  | - Kernel size: 5 × 5  - Feature maps: 20 and 50 | 6 | - | Models in literature | Yes |
| **Custom CNN-based models** | | | | | | |
| [24] | CNN | - | 10 | Tuned | - | - |
| [40] | CNN | - | 15 | Tuned | Models in literature | Yes |
| [47] | CNN | - | - | Tuned | - | - |
| [53] | CNN | - Kernel size:  5 × 5 for separable layers  2 × 2 for pooling layers  - Feature maps: 32, 64, 128, 256 | 4 | Tuned | Models in literature | Yes |
| [57] | CNN | - | 4 | Predefined | - | - |
| [59] | CNN | - Kernel size:  3 × 3 for convolutional layers  2 × 2 for pooling layers | 16 | Tuned | Other shallow models | Yes |
| [61] | CNN | Encoder-decoder network | - | Optimized | Different CNNs | Yes |
| [62] | CNN | - Kernel size:  6 × 6 & 5 × 5 for convolutional layers  2 × 2 & 3 × 3 for pooling layers  - Feature maps: 60 | 4 | Predefined | Number of epochs | - |
| [31] | ResNet | - | 152 | Predefined | Number of layers | - |
| [33] | ResNet | Lesion feature network | 12 | Predefined | Different CNNs | Yes |
| **Hybrid models** | | | | | | |
| [17] | Combined models | Autoencoder and BoF | 2 | Optimized | Standalone  BoF and  autoencoder | Yes |
| [32] | Combined models | multi-target deep convolutional neural network using U-Net and GoogleNet | 18 | Predefined | - | - |
| [38] | Combined models | AlexNet, VGG-16, & ResNet-18 | 8, 16, & 18 | - | Different CNNs | Yes |
| [44] | Combined models | Inception-V3 & Attention | 48 & 1 | Tuned | Different CNNs | Yes |
| [46] | Combined models | - VGG-16 & multi-pooling Attention  - Customer kernel sizes  - 512 feature maps | 22 | Tuned | Different CNNs | Yes |
| **Ensemble models** | | | | | | |
| [20] | Combines 3 ensembles | 1. Single hidden layer BP nets  2. Single hidden layer BP & Fuzzy nets  3. Double hidden layer BP nets | 1, 6, & 6 | Optimized | Other shallow models | Yes |
| [36] | Ensemble | ResNet and Inception V3 | 50 & 48 | Default | Standalone ResNet and Inception V3 | Yes |
| [37] | Ensemble | AlexNet, VGGNet, GoogLeNet | 8, 16, 5, 3, & 22 | Optimized | Different CNNs | Yes |
| [48] | Ensemble | Combined CNNs using fusion | 100 | Tuned | Different depths of ResNets  and  models in ISIC | Yes |
| CNN: convolutional neural networks, ISBI: International Symposium on Biomedical Imaging Challenge, ISIC: International Skin Imaging Collaboration Competition, BP: back propagation, BoF: bag-of-features, “-”: Item not applicable or not discussed in the paper. | | | | | | |
