# Supplementary material for: Artificial Intelligence for Skin Cancer Detection: Scoping Review
Source: J Med Internet Res. 2021 Nov 24;23(11):e22934. doi: 10.2196/22934 (PMC8663507; doi:10.2196/22934)
Supplement: Multimedia Appendix 6 [file jmir_v23i11e22934_app6.docx]

| **Ref** | **Class** | **Dataset  size** | **Model** | **Evaluation Metrics** | | | | | | | | | | |
| --- | --- | --- | --- | --- | --- | --- | --- | --- | --- | --- | --- | --- | --- | --- |
|  |  |  |  | **AC** | **SE** | **SP** | **PR** | **AUC** | **F1** | **DC** | **NPV** | **JI** | **CK** | **YI** |
| [24] | 2 | 40 | CNN | **95.93** | 95.2 | 96.54 | - | - | - | - | - | - | - | - |
| [25] | 2 | 45 | KNN | **86.67** | - | - | - | - | - | - | - | - | - | - |
| [19] | 2 | 80 | SVM | **80** | 71.43 | - | 86.21 | - | - | - | - | - | - | - |
| [10] | 2 | 83 | OpenCV | **66.7** | - | - | - | - | - | - | - | - | - | - |
| [26] | 2 | 100 | SVM | - | 70 | 88 | 86 | **82** | - | - | - | - | - | - |
| [14] | 2 | 170 | CNN | **81** | 81 | 80 | 75 | - | - | - | 86 | - | - | - |
| [11] | 2 | 187 | NB | **89** | 89 | 89 | - | - | - | - | - | - | - | - |
| [15] | 2 | 200 | SVM | - | **90** | **96** | - | - | - | - | - | - | - | - |
| [27] | 2 | 200 | SVM | **97.5** | 97.7 | 96.7 | - | - | 97.5 | - | - | - | - | - |
| [60] | 2 | 200 | SVM | **98.75** | 99.25 | 100 | - | - | - | - | - | - | - | - |
| [20] | 2 | 240 | Ensemble models | **94.17** | 95 | 93.75 | - | - | - | - | - | - | - | - |
| [13] | 2 | 256 | LR | **87.9** | - | - | - | - | - | - | - | - | - | - |
| [28] | 2 | 294 | RF | **77.26** | - | - | - | - | - | - | - | - | - | - |
| [21] | 2 | 356 | SVM | **97.9** | - | - | - | - | - | - | - | - | - | - |
| [29] | 2 | 369 | SVM | - | **76.93** | **69.66** | - | - | - | - | - | - | - | - |
| [12] | 2 | 370 | SVM | - | **83.06** | **90.05** | - | - | - | - | - | - | - | - |
| [30] | 2 | 724 | VGG | **83.51** | 92.57 | 75.39 | 77.14 | 84 | - | - | 91.88 |  | 0.6727 | 67.95 |
| [17] | 2 | 814 | Autoencoder & BoF | **95** | 95.4 | 94.9 | - | - | - | - | - | - | - | - |
| [18] | 2 | 992 | AdaBoost & SVM | **91.73** | 94.08 | 88.71 | - | - | - | - | - | - | 0.831 | - |
| [58] | 2 | 1,031 | DenseNet | **85.5** | - | - | 70 | 84.5 | - | - | - | - | - | - |
| [22] | 2 | 1,250 | ResNet | **94.9** | 91.1 | 95.7 | - | - | - | 89.7 | - | 82.9 | - | - |
| [40] | 2 | 1,276 | CNN | **94.2** | 95 | 94.2 | - | - | - | 94 | - | 93 | - | - |
| [31] | 2 | 1,520 | ResNet | 93 | **96** | **89** | - | 96 | - | 88 | - | - | - | - |
| [59] | 2 | 1,796 | CNN | **75** | 73 | 78 | 77 | - | 75 | - | - | - | - | - |
| [61] | 2 | 2,600 | CNN | **95** | 97 | 96 | - | - | - | 92 | - | - | - | - |
| [42] | 2 | 3,297 | Inception | **90** | - | - | - | - | 87.42 | - | - | - | - | - |
| [39] | 2 | 13,025 | AlexNet | **74.59** | - | - | - | - | - | - | - | - | - | - |
| [50] | 2 | 14,016 | ResNet | 83.9 | 56 | 90.8 | 70.9 | 85 | - | - | - | - | - | - |
| [54] | 2 | 21,659 | ResNet | **82.9** | - | - | - | - | 43.4 | - | - | - | - | - |
| [57] | 2 | 23,907 | CNN | **89.5** | 84 | - | 83.25 | - | 83.25 | - | - | - | - | - |
| [51] | 2 | 48,373 | MobileNet | **91.33** | - | - | - | - | - | - | - | - | - | - |
| [49] | 3 | 429 | ResNet | **87** | - | - | - | - | - | - | - | - | - | - |
| [32] | 3 | 2,000 | U-Net & GoogleNet | - | - | - | - | **88.6** | - | - | - | - | - | - |
| [33] | 3 | 2,000 | ResNet | 85.7 | 49 | 96.1 | 72.9 | **91.2** | - | - | - | - | - | - |
| [37] | 3 | 2,000 | AlexNet, VGGNet, & GoogLeNet | - | - | - | - | **84.8** | - | - | - | - | - | - |
| [41] | 3 | 2,000 | ResNet | - | - | - | - | **91.7** | - | - | - | - | - | - |
| [46] | 3 | 2,750 | Attention & VGG | 86.83 | 57 | 98 | - | **87.12** | - | - | - | - | - | - |
| [48] | 3 | 2,787 | Fusion of CNNs | **87.7** | - | - | - | - | - | - | - | - | - | - |
| [44] | 3 | 3,000 | Attention & Inception | **85.8** | 86 | - | 87 | - | 86 | - | - | - | - | - |
| [16] | 4 | 300 | SVM | **92.96** | - | - | - | - | - | - | - | - | - | - |
| [35] | 5 | 9,144 | AlexNet | **86.21** | - | - | - | - | - | - | - | - | - | - |
| [43] | 5 | 20,000 | Xception | **89** | - | - | - | - | - | - | - | - | - | - |
| [34] | 7 | 8,011 | AlexNet | **84** | 84.7 | 83.8 | - | - | - | - | - | - | - | - |
| [36] | 7 | 10,000 | ResNet & Inception | **89.9** | 79.6 |  | 86.2 | - | - | - | - | - | - | - |
| [38] | 7 | 10,000 | AlexNet, VGG, & ResNet | - | - | - | - | 90.69 | - | - | - | - | - | - |
| [45] | 7 | 10,000 | MobileNet | - | 83 | - | 83 | - | **83** | - | - | - | - | - |
| [47] | 7 | 10,000 | CNN | **75** | - | - | - | - | - | - | - | - | - | - |
| [52] | 7 | 10,000 | VGG | **91.07** | - | - | - | - | - | - | - | - | - | - |
| [55] | 7 | 10,000 | MobileNet | **83.23** | 85 | 87 | - | - | 82 | - | - | - | - | - |
| [56] | 7 | 10,000 | Inception | **72** | - | - | - | - | - | - | - | - | - | - |
| [62] | 7 | 10,000 | CNN | **91** | 85 | - | 87.35 | - | 87.65 | - | - | - | - | - |
| [53] | 7 | 10,000 | CNN | **87.24** | 95.94 | 98.47 | - | - | - | - | - | - | - | - |
| [23] | 9 | 129,450 | Inception | **72.1** | - | - | - | - | - | - | - | - | - | - |
| AC: Accuracy, SE: Sensitivity, SP: Specificity, PR: Precision, AUC: Area under the curve, F1: F1-Score, DC: Dice coefficient, NPV: Negative predictive value, JI: Jaccard index, CK: Cohen’s kappa, YI: Youden’s index, “-”: Metric was not reported in the paper. | | | | | | | | | | | | | | |

## Multimedia Appendix 5: Data, model, and evaluation
